# Entomofauna Associated with Agroforestry Systems of Timber Species and Cacao in the Southern Region of the Maracaibo Lake Basin (Mérida, Venezuela)

**DOI:** 10.3390/insects9020046

**Published:** 2018-04-20

**Authors:** Marina Mazón, Daniel Sánchez-Angarita, Francisco A. Díaz, Néstor Gutiérrez, Ramón Jaimez

**Affiliations:** 1 Biodiversity and Ecosystem Services Research Program, Universidad Nacional de Loja, Ciudadela Universitaria, Sector La Argelia, Loja EC 110101, Ecuador; 2 Departamento de Ciencias Ambientales y Recursos Naturales/Instituto de Investigación de Biodiversidad CIBIO, Universidad de Alicante, Apdo. Correos 99, 03080 Alicante, Spain; 3 Laboratorio de Ciencias Biológicas, Universidad Nacional Experimental de los Llanos Occidentales Ezequiel Zamora, Barinas 3350, Venezuela; dsanchez@unellez.edu.ve; 4 Departamento de Ciencias Biológicas, Universidad Centroccidental “Lisandro Alvarado”, Barquisimeto 3001, Venezuela; fdiaz@ucla.edu.ve; 5 Instituto de Investigaciones para el Desarrollo Forestal, Universidad de Los Andes, Mérida 5101, Venezuela; nestorgutierrez@ula.ve; 6 Instituto de Investigaciones Agropecuarias, Laboratorio Ecofisiología de Cultivos, Universidad de Los Andes, Mérida 5101, Venezuela; rjaimez@utm.edu.ec; 7 Facultad de Ingeniería Agronómica, Universidad Técnica de Manabí, Manabí EC 130105, Ecuador

**Keywords:** native timber trees, Criollo cacao cultivars, Hymenoptera parasitoids, Coleoptera, entomofauna assemblages

## Abstract

Agroforestry systems are environment-friendly production systems which help to preserve biodiversity while providing people with a way of earning a living. Cacao is a historically important crop in Venezuela that traditionally has been produced in agroforestry systems. However, few studies have evaluated how different trees used in those systems affect the dynamics and abundance of insects. The present study evaluated the entomofauna assemblages associated with different combinations of four timber-yielding trees and four Criollo cacao cultivars established in a lowland tropical ecosystem in Venezuela. A randomized block design with two replicates was used, each block having 16 plots which included all 16 possible combinations of four native timber trees (*Cordia thaisiana*, *Cedrela odorata*, *Swietenia macrophylla*, and *Tabebuia rosea*) and four Criollo cacao cultivars (Porcelana, Guasare, Lobatera and Criollo Merideño). Insects were collected with yellow pan traps and sorted to order. Coleoptera and parasitoid Hymenoptera were determined to the family level. In total, 49,538 individuals of seven orders were collected, with Hymenoptera, Diptera, and Hemiptera being the most abundant, although only Lepidoptera and Coleoptera abundances were significantly influenced by the timber tree species. Twenty-three families of parasitoid Hymenoptera and 26 of Coleoptera were found. Significant differences in insects’ assemblages were found both in parasitoid Hymenoptera and Coleoptera families associated to every shade tree, with the families Eulophidae and Lycidae being indicators for *Cordia*, and Chalcididae for *Swietenia*. The entomofauna relationship with the cacao cultivar was barely significant, although Scydmaenidae and Scarabaeidae were indicators for Lobatera and Merideño, respectively. No significant effects were found for interaction with cacao cultivars and native trees. We concluded that the particular insect assemblages found in *Cedrela odorata* and *Cordia thaisiana*, together with their high growing rates, make these two species an optimal choice for cacao agroforestry systems.

## 1. Introduction

Deforestation in Venezuela has greatly increased in the last few decades, with about 326,000 hectares of native forests being destroyed per year for agricultural and livestock purposes with the aim of helping to guarantee the “food and agriculture security” of the country [1]. However, the expectations for production have not been realized, and Venezuela is now suffering the consequences of deforestation, such as more frequent and more severe landslides and a substantial increase in emissions of greenhouse gases [2]. Consequently, there is an urgent need to promote a sustainable farming model that ensures food production and forest conservation in an economically profitable framework for rural communities. Agroforestry, i.e., the combination of trees with crops or livestock [3] may represent an opportunity to achieve these goals. 

Cacao crops have been traditionally cultivated under shade conditions, but presently there is an active debate amongst both scientists and farmers as to whether the cacao crop should be placed under shade conditions or not [4,5,6,7,8]. Physiological traits such as the photosynthetic rates of cacao, which are saturated at low photosynthetic photon flux densities (400–600 μmol photons m^−2^ s^−1^), and low leaf stomatal conductance (g_s_) help explain why cacao is not only a shade-tolerant species [9,10,11,12], but also why it benefits by growing under the shade of other trees [13]. However, monocultures of new sun-grown hybrids are spreading in many tropical countries, especially in those regions where radiation hours and intensity are low [14], resulting in high short-term productivity but with possible long-term implications with regard to the depletion of soils and loss of biodiversity, ultimately affecting the plantation yield [5,6]. Shade varieties may therefore have more advantages than disadvantages [4,7], as long as shade trees contribute additional benefits [15,16], do not compete with cacao for light or nutrients, and are properly managed.

Insects are known to be important in almost all ecological processes within terrestrial ecosystems [17], and their relationship with those ecological processes needs to be established with regard to cultivated lands. This includes both the ecosystem services they provide, such as biological control [18] and pollination [19], and the ecosystem disservice they cause, such as physical and physiological damage produced by herbivorous insects [20,21,22]. However, the implications that different light regimes or the species identity may have on the insect assemblages have been poorly studied. Insects attacking herbivorous insects, such as parasitoid Hymenoptera and some predator beetles, while helping to balance the agroecosystem by natural control, are more sensitive to disturbances than their hosts [23,24]. However, it is unclear whether shade trees in cacao agroforestry systems induce pest outbreaks or provide a useful ecosystem service by increasing their natural enemies’ populations [25,26,27,28].

The Venezuelan Criollo cacao cultivars (*Theobroma cacao* L.) have exquisite flavours and aromas [29,30], and are highly appreciated by the chocolate industry. Nevertheless, they have low fruit production and are highly sensitive to pests and diseases, which make them of little interest economically to the local farmers. Hence, these cultivars have been replaced by more resistant Trinitario- and Forastero-type cultivars and as a consequence, Criollo cacaos are endangered. However, in Venezuela, since the beginning of 2000, several national projects have been promoted in order to rescue the high-yielding Criollo cultivars by increasing their production and gradually introducing them on cacao plantations. Managing the cacao cultures in order to favour pest natural control by favouring the occurrence of their potential natural enemies may also help to recover these traditional cultivars [27,31].

In the present study we assessed the insect assemblages associated to four native timber trees in a cacao agroforestry system by using two indicator groups: Coleoptera and parasitoid Hymenoptera, since both groups have been demonstrated to be very sensitive to changes in the environment [32,33,34,35]. We used a higher-level taxa for the tests, i.e., family level. Family richness can be used as a surrogate for species richness, since it is closely correlated to species richness in some groups [36,37]. The aims of this study were: (1) to assess insect richness and abundance at different taxonomic levels in the agroforestry system of Criollo cacao cultivars with native timber trees; and (2) to evaluate the influence of the native timber trees and the cacao cultivars over the insect assemblages associated. 

## 2. Materials and Methods

### 2.1. Study Area

The study was conducted at “The Judibana” farm, Universidad de Los Andes, in the Municipality Alberto Adriani, Mérida state, Venezuela Western region (8°37′26″ N and 71°42′22″ W), at an altitude of 73 m.a.s.l. The temperature, relative humidity and rainfall annual average were 28.6 °C, 77.7% and 1834 mm, respectively (Venezuela Air Force, station El Vigia Airport). Previous studies [20] reported that soils ranged between sandy loam and sandy, with pH between 5.2 and 6.3, N content between 0.03% and 0.1%, P between 2 and 8 mg/kg, and K between 25 and 118 mg/kg.

### 2.2. Sampling and Determination

**Figure 1:**
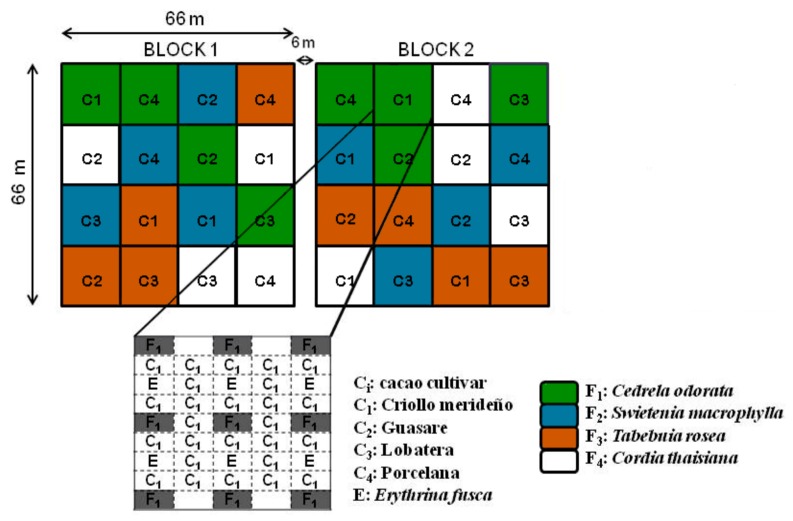
Diagram representing the blocks of treatments at Judibana farm. The lower figure shows spatial distribution of timber trees and cacao plants in each plot (modified from Jaimez et al. [20]).

The study was carried out in agroforestry systems that combined timber tree with cacao cultivars. These agroforestry systems were established in an area previously used for livestock whose vegetation was composed of pastures and some non-commercial native tree species randomly distributed. Prior to plantation, the vegetation of the area was eliminated and all trees were planted between January and March of 2007 in a random block design with three replicates (i.e., three blocks) in an area of two ha, although only two blocks were actually sampled since several plants from block three were damaged in the early stages (Figure 1). Each block had 16 plots (24 × 9 m) that corresponded to randomized combinations of one of the four timber species with one of the four cacao cultivars, meaning that there were two plots (one per block) with each of the 16 possible combinations of every timber tree and each cultivar. Additionally all plots had the tree legume species *Erythrina fusca* Lour (Fabaceae). Each plot was thus composed of 15 trees, i.e., nine timber-yielding trees and six *E. fusca* trees, planted 6 m apart, along with 26 cacao plants (Figure 1). The 26 cacao plants were planted in rows with alternating lines of timber trees and *E. fusca*. Cacao plants were separated by a distance of 3 × 3 m. A more detailed description of the experiment design is given in Jaimez et al. [20].

The four timber species employed were: *Cordia thaisiana* Agostini (Boraginaceae), *Cedrela odorata* L. (Meliaceae), *Swietenia macrophylla* King (Meliaceae) and *Tabebuia rosea* (Bertol) A.D.C. (Bignoniaceae) (from here on, *Cordia*, *Cedrela*, *Swietenia* and *Tabebuia*, respectively), which have varying characteristics (Table 1). *Cordia* and *Swietenia* are commonly used in commercial monospecific plantations, while *Cedrela* and *Tabebuia* are usually planted in random combinations with other tree species (fruits and legume species) for cocoa shade. In the study region, *Cedrela* is a common tree in most cacao plantations. All species are used for the construction of furniture, handicrafts and boats, as well as for household decoration, and are under special protection regimes due to their large timber extraction rates. These species are in high demand by the national timber industry and are species that local farmers are familiar with.

The native Venezuelan Criollo cacao cultivars were: Porcelana, Guasare, Lobatera and Criollo Merideño. In the Western region of Venezuela, farmers have traditionally kept in their cacao plantations a mixture of Criollo and Forastero types. The Porcelana cultivar has been maintained for more than a century in the area because of its high quality, and is highly valued in European cacao factories. The cultivar Guasare was rescued from the Venezuelan Guajira region in the 1990s and has been distributed to farmers by the Venezuelan National Institute of Agricultural Research (INIA). The two other cultivars (Criollo Merideño and Lobatera) have been recently selected for their productivity from within breeding programs carried out by the INIA. Criollo Merideño is found in regions above 200 m.a.s.l. Guasare, Criollo Merideño and Lobatera were selected due to their high productive potential, greater than that of the Porcelana cultivar which has been traditionally used in the region.

By the time insects were sampled, the vegetation around the agroforestry system was composed of abandoned pastures with little livestock intervention. The whole plantation was covered by a 20-cm-high uniform herbaceous layer. Insects were collected by using 20-cm-long and 8-cm-deep square yellow pan traps, filled with a solution of salt saturated water with a few drops of detergent to reduce surface tension. Yellow pan traps were selected since they are discreet, easy to manipulate, and have been widely used for collecting various groups of insects, particularly parasitoid Hymenoptera [38], and also other vegetation-related insects, especially when placed on the ground [39,40]. Three traps were placed forming a triangle, separated 30 cm each, in the middle of every plot at ground level, and insects collected by the three traps in the same plot were pooled as a single sample. Sampling took place during the wet season, from September 2011 to January 2012, with monthly rainfall over 150 mm with a peak of 250 mm in November (Venezuela Air Force, station El Vigía Airport). Traps were operating over six consecutive days each month, with the content being replaced every three days to avoid decomposition of the samples. When replacing, material was washed in abundant water and transferred to a pot filled with 70% ethanol.

All adult individuals collected were sorted to insect orders and counted. Specimens belonging to parasitoid Hymenoptera and Coleoptera were identified to family level. Other families occurring in high abundances and easily recognizable were also separated and counted.

All determined specimens are stored in the Colección del Laboratorio de Ecología de Insectos (CLEI) at the Universidad de Los Andes (Mérida, Venezuela).

### 2.3. Data Analysis

Abundance (number of individuals) and richness (number of taxa) were calculated at two taxonomic levels: orders (considering all of them) and families (including only families belonging to Coleoptera and Hymenoptera parasitoids). Abundance (number of individuals) was evaluated for all the individuals collected, and separately for every order. These two variables were tested for normal distribution and for equality of variances by means of Shapiro–Wilk and Levene’s tests, respectively. Data did not fit a normal distribution (*p* < 0.000005), but variances were equal (*p* > 0.05), both for means and medians, so we performed an ANOVA test (one and two ways) to compare insect assemblages for the four species of native trees, the four cultivars and the combination of both. 

Mardia’s test was performed for multivariate normality, and since data did not fit a normal distribution (S = 4722, *p* = 2.56 × 10^−216^ and S = 12,300, *p* = 0, for Hymenoptera and Coleoptera families, respectively) then differences in families’ assemblages were evaluated by means of a two-way Permutational multivariate analysis of variance (PERMANOVA) test. This non parametric multivariate test compares the location between group centroids of assemblages by using different variables [41]. Before the analysis, data were square-root transformed, and Bray–Curtis distances were used. The explanatory variables were the type of native timber tree and the cacao cultivars. The dependent variables were abundances of families of parasitoid Hymenoptera and Coleoptera. When results from PERMANOVA were significant, a similarity percentage (SIMPER) test was done in order to know which taxa were responsible for these differences [42]. Additionally, we conducted an indicator species analysis (ISA) to assess the value of the families as an indicator of any of the factors considered.

ANOVA, PERMANOVA, and SIMPER tests, as well as the normality test, were done with software Past [43], boxplots were performed using Statistica version 7.0 (Stat Soft, Inc., Tulsa, OK, USA), and the ISA was run with PC-ORD 7.03 [44].

## 3. Results

We collected 49,538 individuals, belonging to seven orders: Orthoptera, Lepidoptera, Hymenoptera, Hemiptera, Diptera, Coleoptera, and Dictyoptera. The orders Hymenoptera, Diptera and Hemiptera were the most abundant orders, comprising 95% of the total insects with 19,118, 13,363 and 14,550 individuals collected, respectively.

**Figure 2:**
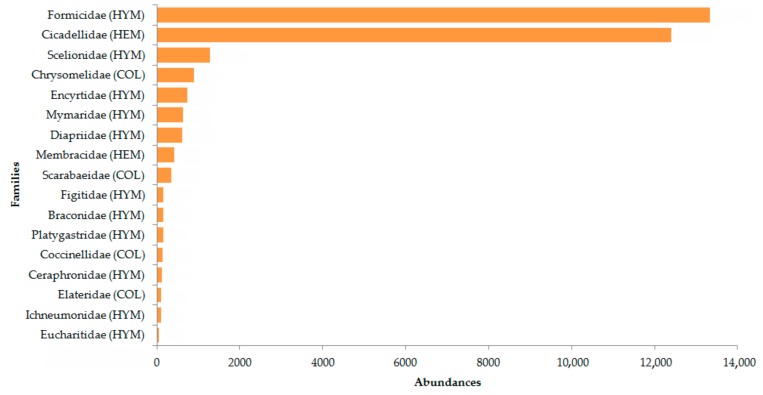
Abundance of the major insect families (*n* > 50 individuals) found in the samples. Abbreviations of the order they belong to are in brackets: HYM = Hymenoptera; HEM = Hemiptera; COL = Coleoptera.

We found 23 families of parasitoid Hymenoptera (Table 2) and 24 of Coleoptera (Table 3). In Figure 2, the most abundant families (*n* > 50 individuals) are shown. Formicidae and Cicadellidae were included in the count because they were extremely abundant within the samples, representing more than 80%.

### Influence of Native Timber Tree Species and Cacao Cultivars on Entomofauna

**Figure 3:**
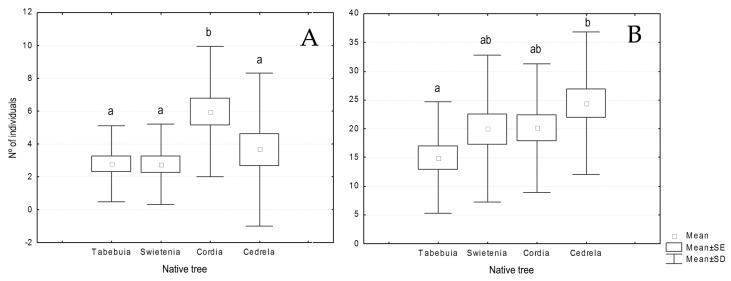
Mean abundances of Lepidoptera (**A**) and Coleoptera (**B**) associated to every type of timber tree. Different letters show significant differences (*p* < 0.05).

No significant differences were found between total abundance nor richness of orders occurring associated to every timber tree (F = 0.119, *p* = 0.949 and F = 0.974, *p* = 0.409, respectively), nor to every cacao cultivar (F = 0.483, *p* = 0.695 and F = 0.487, *p* = 0.692, respectively), nor the interaction of both (F = 0.909, *p* = 0.522, and F = 0.812, *p* = 0.607). However, when analysing every order separately, we found that Lepidoptera and Coleoptera abundances were influenced by the tree species, and Lepidoptera by the cacao cultivar also. While Lepidoptera was significantly (F = 4.77, *p* = 0.004) more abundant in *Cordia* plots than in *Tabebuia* and *Swietenia* (Figure 3A), Coleoptera was significantly (F = 2.598, *p* = 0.050) more present in *Cedrela* than in *Tabebuia* plots (Figure 3B). Regarding the cacao cultivar, Lepidoptera was significantly more abundant in Porcelana plots (F = 4.066, *p* = 0.009). 

Regarding family richness (including all Coleoptera and parasitoid Hymenoptera families), no significant differences between the different native trees were found (F = 0.751, *p* = 0.525), nor with the interaction of timber tree and cacao cultivar (F = 0.868, *p* = 0.557), but the Merideño cultivar had a significantly higher family richness than Guasare (F = 3.694, *p* = 0.015). 

The PERMANOVA analysis showed significant differences in both Hymenoptera and Coleoptera assemblages associated to the native timber trees (F = 1.792, *p* = 0.001 and F = 1.728, *p* = 0.024, respectively). When comparing cacao cultivar, there were significant effects only on the Coleoptera assemblages (F = 1.995, *p* = 0.005), and none of the interactions between cacao cultivars and native trees were significant, indicating the independence of these factors. Considering parasitoid Hymenoptera family assemblages, those occurring in *Cedrela* plots were significantly different to the *Swietenia* and *Tabebuia* ones, as well as those in *Cordia* when compared to those in *Swietenia* (Table 4). The families Diapriidae, Scelionidae, Encyrtidae, Mymaridae and Platygastridae explained about 50% of these differences: they were relatively less abundant in *Cedrela* than in the other trees, whilst Diapriidae and Encyrtidae were more abundant in *Cordia* than in *Swietenia* (see Supplementary Materials Table S1); however, only the families Eulophidae and Chalcididae were considered significant as indicator species for *Cordia* and *Swietenia* trees species, respectively (Table 5). 

On the other hand, considering Coleoptera families’ assemblages, significant differences were found between *Tabebuia* and *Cordia* (Table 4). Chrysomelidae, Scarabaeidae, Coccinellidae, Elateridae, and Curculionidae contributed the most, explaining 62% of the differences. Most families were more abundant in *Cordia* plots than in those with *Tabebuia*, while only Scarabaeidae and Curculionidae were more present in *Tabebuia* than in *Cordia* (see Supplementary Materials Table S2). The family Lycidae was a significant indicator for *Cordia* trees, as was Biphyllidae for *Cedrela* (Table 6). There were also significant differences in the Coleoptera assemblages amongst cacao cultivars: those occurring in Guasare plots were different to Lobatera and Merideño. Chrysomelidae, Scarabaeidae, Coccinellidae, Elateridae, Scydnaenidae, Curculionidae, and Staphylinidae contributed to 70–77% of the total dissimilarity, they were less abundant in Guasare than in the other cultivars (see Supplementary Materials Table S3). Scydmaenidae and Scarabaeidae turned out to be significant indicator families for Lobatera and Merideño, respectively (Table 7).

## 4. Discussion

The entomofauna associated with the agroforestry system studied included the major insect orders, with samples being dominated by Hymenoptera, Hemiptera, and Diptera. Ants (Formicidae) and leafhoppers (Cicadellidae) were by far the most abundant groups. The dominance of these groups was influenced by the type of trap used, since it was placed on the ground and by consequence it mainly collected ground insects or those living in the herbaceous and litter layers. Coloured pan traps are considered a standardized methodology for comparison of Hymenoptera diversity amongst sites [45,46] and for collecting some Coleoptera families [47]. The high abundance of ants may be favoured by the presence of *Cordia* trees, since a close symbiotic relationship has been described between ants and some *Cordia* species [48]. Jaimez et al. [20] found that *Cordia* trees were not attacked by insects in this plantation, while *Tabebuia* and *Swietenia* were highly susceptible to ant attacks during the first two years of establishment. In our study, Formicidae abundance was not significantly higher in *Cordia* plots, although that may be explained by the young age of the trees, since one year after sampling this relationship was more noticeable [20]. Besides Formicidae and Cicadellidae, yellow traps, as shown in this study, are effective for collecting Proctotrupoidea and Platygastroidea [49], as well as some Chalcidoidea families [50], while other insect traps commonly used, like malaise traps, usually collect greater amounts of Ichneumonoidea [38,45,49,50,51].

Concerning Hymenopteran parasitoids, we found 23 of the 44 Neotropical families belonging to the superfamilies mentioned above [52]. This is of low richness compared to the 33 families of parasitoid Hymenoptera found in different traditional cacao plantations of the same Venezuelan state [53], but significantly higher than the 11 Hymenoptera families collected in a monospecific silvopastoral system of *Brachiaria decumbens* in Minas Gerais [50]. Furthermore, the richness of parasitoid families recorded in this study was similar to that found in some Amazonian forest reserves [51], and their relative abundances are consistent with those found by [45] by means of yellow pan traps at the edge of a rainforest in Sulawesi. 

Many of the hymenopteran families we found in this survey, specifically Ichneumonidae, Braconidae, Eurytomidae, Chalcididae, Trichogrammatidae, Encyrtidae, Aphelinidae, Eulophidae, Eupelmidae, and Signiphoridae, have been recorded as parasitoids of phytophagous species that feed on cacao [54]. Although we did not identify the individuals to species level, both abundance and family richness of parasitoid Hymenoptera are good predictors of the richness of other taxa [55]. The occurrence of these families within the farm, while indicating relatively high overall biodiversity, may also be contributing to long-term sustainability due to the potential regulation of phytophagous insect assemblages, since parasitoids can keep pest populations at low densities [56] while generating responses at a radius of hundreds of meters [57]. However, the high incidence of *Hypsipyla grandella* (Zeller) attacking mainly *Cedrela* in the Judibana plantation during the early stages [20], which is one of the main pests of *Cedrela* and *Swietenia* in Venezuela [58], may indicate the absence of a suitable population of their native parasitoids. One Eulophidae species, *Palmistichus elaeisis* (Delvare & LaSalle), has been reported as parasitizing *H. grandella* pupae in Brazil [59], and actually this family was more abundant in the *Cedrela* plots than in others, but it may not be present enough. In *Tabebuia*, the pyralid *Eulepte gastralis* (Gn.) may be a severe defoliator in young plantations, especially when sowing density is high [60], but in 2–3 years plantations, leaf-rolling insects tend to be the most harmful [61]; none of them were reported in Judibana plantation, or at least not being economically important [20].

We also found a high richness of Coleoptera families, which represents a high diversity of trophic guilds too, contributing to the maintenance of several ecosystem services (e.g., natural pest control, nutrients recycling). The most abundant families, i.e., Chrysomelidae and Scarabaeidae, coincide with those found on the lower canopy level of some cacao agroforestry systems in Indonesia [62]. Some species of chrysomelids have been recorded from cocoa plants [63], so it is important to make identifications at genus or species level in this family. Besides, the relatively low abundance of Scolytinae subfamily suggests a more or less healthy farm, compared for example to a sampling made in another Venezuelan Criollo cacao plantation, where about 20 Scolytinae were collected per trap [22]. This group may constitute one of the most important pests in cacao by helping to spread many phytopathogenic fungi [64,65], which have been recorded as the main concern for cacao farmers in Mérida plantations [22] and have also been associated with the shade trees of cacao agroforestry systems, such as *Swietenia* and *Tabebuia* [66]. However, the lack of complementary collecting methods, such as alcohol traps, light traps, and direct capture, in addition to the seasonal sampling, may be affecting this result [66]. The occurrence of families such as Coccinellidae, Staphylinidae, and Carabidae indicates a community of generalist predator beetles that could be effective at controlling populations of herbivores such as Chrysomelidae, Scarabaeidae, and Elateridae, whereas the low abundance of bark beetle predators such as Histeridae could be related to the low abundance of prey [67].

The type of timber tree shade may determine the presence or absence of determined species due to effects on thermoregulation [68]. A moderate shade seems to benefit some insects, like Scarabaeidae dung beetles or parasitic wasps [26], but implications of shade management over Coleoptera diversity are unclear [62]. In the study area, Coleoptera were significantly more abundant in *Cedrela* than in *Tabebuia*, both with similar shade cast. It seems that, independently of the shade cast, the identity of the trees do determine Coleoptera preferences [69], and may influence the preference of a determined herbivorous pest to an herbaceous host plant [70].

*Cordia* timber species have been recommended for coffee plantations because of their potentially sustainable output of timber [16,71]. *Cedrela* may constitute a suitable option as a shade tree in coffee plantations, provided that it does not compete with coffee plants [72], although the survival rate may be low during the first years after plantation [73]. *Cordia* and *Cedrela* showed the best growing rates during the first three years in the studied plantation [20,74], and gathered more abundant and significantly different insect assemblages according to the present work. Besides, their shade cast is consistent with Beer’s recommendation [4]: a “regular mottled shade pattern” in order to not reduce the light quality that would be available for the cacao trees. 

The limited relationship between the native timber trees and the characteristics of the insect assemblages tested in this study may indicate that the local habitat variables do not have a great impact on these groups with relatively high mobility. Instead, landscape variables may have more weight to explain their diversity and abundance, as shown for different insect groups [35,75,76,77]. Mixed-species plantations of timber trees may reduce the herbivore damage [78], although this will depend on the particular pest species, since mixed plantations may favour or be detrimental to them by the opposite resource concentration and dilution effects, as seen for *Tabebuia* plantations [78]. Furthermore, the presence of non-host herbivores may reduce the efficiency of several natural enemies by increasing the time they have to spend to find their specific hosts [79].

Despite the limitations of the sampling design, this study gives a general picture of the overall insect diversity associated with some of the timber species that may be used as shade trees in cacao plantations. The trap type we used may show a bias in the sampled groups, so further studies should be done in order to know which species are directly related to the timber species and how they may effectively help to improve productivity of cacao plantations, and, as a consequence, ensure the long-term livelihoods of cacao farmers.

## Figures and Tables

**Table 1:** Distribution, functional type (leaf phenology), and tree height of four timber species.

Species	Distribution	Functional Type	Height (m)
*Cedrela odorata*	Northern Mexico to Northern Argentina	Deciduous	40
*Swetenia macrophylla*	Southern Mexico, Central American countries (from Belize to Panama) and South America in Venezuela, Colombia and the Amazon of Peru and Brazil	Semi deciduous	35–45
*Tabeuia rosea*	Southern Mexico, Central America, reaching Ecuador in South America	Semi deciduous	20–30
*Cordia thaisiana*	South of Mexico and Panama, Brazil, Colombia and Venezuela	Evergreen	20

**Table 2:** Abundances of the families of parasitoid Hymenoptera collected in all samples.

Superfamily	Family	Abundance
Ceraphronoidea	Ceraphronidae	116
Chalcidoidea	Agaonidae	7
	Aphelinidae	5
	Chalcididae	31
	Encyrtidae	741
	Eucharitidae	51
	Eulophidae	22
	Eupelmidae	7
	Eurytomidae	6
	Mymaridae	637
	Pteromalidae	57
	Signiphoridae	2
	Trichogrammatidae	9
Chrysidoidea	Bethylidae	28
	Chrysididae	4
	Dryinidae	14
Cynipoidea	Figitidae	157
Evanioidea	Evaniidae	3
Ichneumonoidea	Braconidae	152
	Ichneumonidae	96
Platygastroidea	Scelionidae	1275
	Platygastridae	150
Proctotrupoidea	Diapriidae	618

**Table 3:** Abundances of families of Coleoptera collected in all samples. Main trophic guilds of every family are included.

Superfamily	Family	Abundance	Trophic Guild
SubO. Adephaga			
Caraboidea	Carabidae	6	Generalist predator
	Cicindelidae	2	Generalist predator
	Dytiscidae	3	Generalist predator
SubO. Polyphaga			
Chrysomeloidea	Chrysomelidae	897	Herbivore
Cucujoidea	Biphyllidae	17	Detritivore
	Coccinellidae	132	Generalist predator
	Erotylidae	4	Detritivore
	Rhizophagidae	1	Bark beetle predator/Detritivore
Curculionoidea	Curculionidae	78	Herbivore
	(Subfamily Scolytinae	32)	Bark borer
Elateroidea	Elateridae	99	Herbivore
	Lampyridae	1	Generalist predator
	Lycidae	8	Detritivore/Herbivore
Hydrophiloidea	Histeridae	6	Bark beetle predator
Scarabaeoidea	Aphodiidae	31	Detritivore
	Hybosoridae	7	Detritivore
	Rutelidae	3	Herbivore
	Scarabaeidae	352	Herbivore
Staphylinoidea	Pselaphidae	5	Detritivore
	Scydmaenidae	29	Generalist predator
	Staphylinidae	43	Generalist predator
Tenebrionoidea	Anthicidae	6	Omnivore
	Colydiidae	4	Detritivore
	Mordellidae	16	Herbivore
	Mycetophagidae	1	Detritivore

**Table 4:** *p*-values obtained from PERMANOVA analysis when comparing both parasitoid Hymenoptera families assemblages (above the diagonal) and Coleoptera families assemblages (below the diagonal) associated to every native tree species in the study. In italics when significant (*p* < 0.05).

	*Cedrela*	*Cordia*	*Swietenia*	*Tabebuia*
***Cedrela***	0	0.1759	*0.0274*	*0.0394*
***Cordia***	0.0863	0	*0.0066*	0.0905
***Swietenia***	0.8157	0.0891	0	0.5315
***Tabeuia***	0.0834	*0.0023*	0.4700	0

**Table 5:** Results obtained from the indicator species analysis in parasitoid Hymenoptera families associated to every native tree species in the study. In italics when significant (*p* < 0.05). IndVal = indicator value.

Families	Tree Species	IndVal	*p*
Eulophidae	*Cordia*	*21.7*	*0.0112*
Chalcididae	*Swietenia*	*20.1*	*0.0289*
Diapriidae	*Cordia*	29.4	0.0922
Pteromalidae	*Cordia*	19.1	0.1022
Figitidae	*Tabebuia*	*26.6*	0.1250
Ichneumonidae	*Cordia*	21.0	0.1418
Encyrtidae	*Tabebuia*	28.4	0.1842
Bethylidae	*Cedrela*	12.3	0.2150
Scelionidae	*Swietenia*	27.7	0.2216
Dryinidae	*Cedrela*	9.5	0.2360
Eurytomidae	*Swietenia*	6.9	0.3885
Platygastridae	*Swietenia*	22.1	0.3995
Ceraphronidae	*Cordia*	19.3	0.5053
Braconidae	*Swietenia*	22.3	0.6005
Aphelinidae	*Cordia*	5.3	0.6077
Trichogrammatidae	*Swietenia*	4.8	0.8266
Eucharitidae	*Cordia*	10.4	0.8620
Mymaridae	*Cordia*	25.8	0.8776
Chrysididae	*Tabebuia*	4.2	0.9040
Eupelmidae	*Cordia*	2.9	0.9726
Agaonidae	*Cedrela*	2.4	1.00
Signiphoridae	*Cordia*	2.1	1.00
Evaniidae	*Cedrela*	1.4	1.00

**Table 6:** Results obtained from the indicator species analysis in Coleoptera families associated to every native tree species in the study. In italics when significant (*p* < 0.05). IndVal = indicator value.

Families	Tree Species	IndVal	*p*
Chrysomelidae	*Cordia*	29.4	0.0508
Biphyllidae	*Cedrela*	*17.7*	*0.0336*
Coccinellidae	*Cedrela*	22.6	0.2106
Erotylidae	*Cordia*	4.2	0.9016
Rhizophagidae	*Cedrela*	*4.2*	1.00
Curculionidae	*Cedrela*	14.6	0.4799
Elateridae	*Cordia*	15.4	0.6677
Lampyridae	*Cedrela*	4.2	1.00
Lycidae	*Cordia*	*14.0*	*0.0340*
Histeridae	*Tabebuia*	3.7	0.5975
Aphodiidae	*Tabebuia*	13.0	0.0762
Scarabaeidae	*Cedrela*	29.9	0.0568
Hybosoridae	*Cedrela*	4.0	0.7746
Rutelidae	*Cedrela*	5.6	0.6043
Pselaphidae	*Swietenia*	3.8	0.8970
Scydmaenidae	*Cedrela*	7.3	0.9714
Staphylinidae	*Tabebuia*	10.8	0.2747
Anthicidae	*Cedrela*	3.1	1.00
Mordellidae	*Cordia*	7.4	0.4585
Colydiidae	*Cordia*	1.7	1.00
Mycetophagidae	*Swietenia*	4.2	1.00
Carabidae	*Swietenia*	2.8	1.00
Cicindelidae	*Tabebuia*	8.3	0.2521
Dytiscidae	*Tabebuia*	8.3	0.2372

**Table 7:** Results obtained from the indicator species analysis in Coleoptera families associated to every cacao cultivar in the study. In italics when significant (*p* < 0.05). IndVal = indicator value.

Families	Cacao Cultivar	IndVal	*p*
Chrysomelidae	Porcelana	29.0	0.0880
Biphyllidae	Lobatera	9.5	0.4465
Coccinellidae	Merideño	20.6	0.4069
Erotylidae	Guasare	4.2	0.9006
Rhizophagidae	Merideño	*4.2*	1.00
Curculionidae	Merideño	13.6	0.5849
Elateridae	Merideño	16.2	0.5475
Lampyridae	Lobatera	4.2	1.00
Lycidae	Lobatera	3.3	0.8774
Histeridae	Porcelana	3.1	0.8544
Aphodiidae	Porcelana	5.7	0.6835
Scarabaeidae	Merideño	*30.6*	*0.0314*
Hybosoridae	Merideño	4.0	0.7766
Rutelidae	Lobatera	1.4	1.00
Pselaphidae	Guasare	4.6	0.6181
Scydmaenidae	Lobatera	*22.2*	*0.0138*
Staphylinidae	Porcelana	8.0	0.6455
Anthicidae	Merideño	6.9	0.3321
Mordellidae	Merideño	8.9	0.3147
Colydiidae	Merideño	5.9	0.4295
Mycetophagidae	Merideño	4.2	1.00
Carabidae	Porcelana	11.1	0.1332
Cicindelidae	Lobatera	2.1	1.00
Dytiscidae	Merideño	8.3	0.5944

## Supplementary Materials

The following are available online at http://www.mdpi.com/2075-4450/9/2/46/s1, Table S1: Results obtained from SIMPER analysis when comparing those pairs of parasitoid Hymenoptera families assemblages with significant differences (*p* < 0.05). Table S2: Results obtained from SIMPER analysis when comparing those pairs of Coleoptera families assemblages with significant differences (*p* < 0.05) amongst timber trees. Table S3: Results obtained from SIMPER analysis when comparing those pairs of Coleoptera families assemblages with significant differences (*p* < 0.05) amongst cacao cultivars.

## References

[1] Kim D.H., Sexton J.O., Townsend J.R. (2015). Accelerated deforestation in the humid tropics from the 1990s to the 2000s. Geophys. Res. Lett..

[2] MINEA, Ministerio del Poder Popular para el Ecosocialismo y Aguas (2017). Segunda Comunicación Nacional Ante la Convención Marco de las Naciones Unidas Sobre Cambio Climático de Venezuela.

[3] Stamps W.T., Linit M.J. (1998). Plant diversity and arthropod communities: Implications for temperate agroforestry. Agrofor. Syst..

[4] Beer J. (1987). Advantages, disadvantages and desirable characteristics of shade trees for coffee, cacao and tea. Agrofor. Syst..

[5] Belksy J.M., Siebert S.F. (2003). Cultivating cacao: Implications of sun-grown cacao on local food security and environmental sustainability. Agric. Hum. Values.

[6] Ruf F.O. (2011). The myth of complex cocoa agroforests: The case of Ghana. Hum. Ecol..

[7] Abou Rajab Y., Leuschner C., Barus H., Tjoa A., Hertel D. (2016). Cacao cultivation under diverse shade tree cover allows high carbon storage and sequestration without yield losses. PLoS ONE.

[8] Famuwagun I.B., Agele S.O., Aiyelari O.P. (2018). Shade effects on growth and development of cacao following two years of continuous dry season irrigation. Int. J. Fruit Sci..

[9] Almeida A.A.F., Gomes F., Araujo R., Santos R.C., Valle R. (2014). Leaf gas exchange in species of the *Theobroma* genus. Photosynthetica.

[10] Ávila-Lovera E., Coronel I., Jaimez R., Urich R., Pereyra P., Araque O., Chacón L., Tezara W. (2016). Ecophysiological traits of adult trees of Criollo cacao cultivars (*Theobroma cacao* L.) from a germplasm bank. Exp. Agric..

[11] Araque O., Jaimez R., Tezara W., Coronel I., Urich R., Espinosa W. (2012). Comparative photosynthesis, water relations, growth and survival rates in juvenile Criollo cacao cultivars (*Theobroma cacao*) during dry and wet seasons. Exp. Agric..

[12] Acheampong K., Hadley P., Daymond A.J. (2013). Photosynthetic activity and early growth of four cacao genotypes as influenced by different shade regimes under West African dry and wet season conditions. Exp. Agric..

[13] Somarriba E., Beer J. (2011). Productivity of *Teobroma cacao* agroforestry systems with timber and legume service shade tree. Agrofor. Syst..

[14] Jaimez R.E., Amores Puyutaxi F., Vasco A., Loor R.G., Tarqui O., Quijano G., Jimenez J.C., Tezara W. (2018). Photosynthetic response to low and high light of cacao growing without shade in an area of low evaporative demand. Acta Biol. Colomb..

[15] Tscharntke T., Shonil Y., Bhagwat S., Buchori D., Faust H., Hertel D., lscher D.H., Juhrbandt J., Kessler M., Perfecto I. (2011). Multifunctional shade-tree management in tropical agroforestry landscapes—A review. J. Appl. Ecol..

[16] Somarriba E., Suárez-Islas A., Calero-Borge W., Villota A., Castillo C., Vílchez S., Deheuvels O., Cerda R. (2014). Cocoa-timber agroforestry systems: *Theobroma cacao*-*Cordia alliodora* in Central America. Agrofor. Syst..

[17] Dunn R.R. (2005). Modern insect extinctions, the neglected majority. Conserv. Biol..

[18] Duelli P., Obrist M.K. (1998). In search of the best correlates for local organismal biodiversity in cultivated areas. Biodivers. Conserv..

[19] Adjaloo M.K., Oduro W. (2013). Insect assemblage and the pollination system of cocoa (*Theobroma cacao* L.). J. Appl. Biosci..

[20] Jaimez R.E., Araque O., Guzman D., Mora A., Espinoza W., Tezara W. (2013). Agroforestry systems of timber species and cacao: Survival and growth during the early stages. J. Agric. Rural Dev. Subtrop..

[21] Barrios K., Mazón M., Chacón M.M., Otero L.D., Gaviria J. (2012). Comunidad de lepidópteros asociados a *Theobroma cacao* L. en agroecosistemas con diferente manejo de sombra (Mérida, Venezuela). Ecotrópicos.

[22] Mazón M., Díaz F., Gaviria J. (2013). Effectiveness of different trap types for control of bark and ambosia beetles (Scolytinae) in Criollo cacao farms of Mérida, Venezuela. Int. J. Pest Manag..

[23] Thies C., Steffan-Dewenter I., Tscharntke T. (2003). Effects of landscape context on herbivory and parasitism at different spatial scales. Oikos.

[24] Holzschuh A., Steffan-Dewenter I., Tscharntke T. (2010). How do landscape composition and configuration, organic farming and fallow strips affect the diversity of bees, wasps and their parasitoids?. J. Anim. Ecol..

[25] Beer J., Muschler R., Kass D., Somarriba D. (1998). Shade management in coffee and cacao plantations. Agrofor. Syst..

[26] Rao M.R., Singh M.P., Day R. (2000). Insect pest problems in tropical agroforestry systems: Contributory factors and strategies for management. Agrofor. Syst..

[27] Bianchi J.J.A., Booij C.J.H., Tscharntke T. (2006). Sustainable pest regulation in agricultural landscapes: A review on landscape composition, biodiversity and natural pest control. Proc. R. Soc. Lond. B.

[28] Novais S.M.A., Macedo-Reis L.E., DaRocha W.D., Neves F.S. (2016). Effects of habitat management on different feeding guilds of herbivorous insects in cacao agroforestry systems. Rev. Biol. Trop..

[29] Counet C., Ouwerx C., Rosoux D., Collin S. (2004). Relationship between procyanidin and flavor contents of cocoa liquors from different origins. J. Agric. Food Chem..

[30] Zambrano A., Gomez A., Ramos G., Romero C., Lacruz C., Rivas E. (2010). Caracterización de parámetros físicos de calidad en almendras de cacao criollo, trinitario y forastero durante el proceso de secado. Agron. Trop..

[31] Bullock J.M., Aronson J., Newton A.C., Pywell R.F., Rey-Benayas J.M. (2011). Restoration of ecosystem services and biodiversity: conflicts and opportunities. Trends Ecol. Evol..

[32] LaSalle J., Gauld I.D. (1993). Hymenoptera and Biodiversity.

[33] Favila M., Halffter G. (1997). The use of indicator groups for measuring biodiversity as related to community structure and function. Acta Zool. Mex..

[34] Escobar F., Halffter G., Solís A., Halffter V., Navarrete D. (2008). Temporal shifts in dung beetle community structure within a protected area of tropical wet forest: A 35-year study and its implications for long-term conservation. J. Appl. Ecol..

[35] Verdú J.R., Numa C., Hernández-Cuba O. (2011). The influence of landscape structure on ants and dung beetles diversity in a Mediterranean savanna-Forest ecosystem. Ecol. Indic..

[36] Balmford A., Green M.J.B., Murray M.G. (1996). Using higher-taxon richness as a surrogate for species richness: I. Regional tests. Proc. R. Soc. Lond. B.

[37] Bragança M.A.L., Zanuncio J.C., Picanço M., Laranjeiro A.L. (1998). Effects of environmental heterogeneity on Lepidoptera and Hymenoptera populations in Eucalyptus plantations in Brazil. For. Ecol. Manag..

[38] Mazón M., Bordera S. (2008). Effectiveness of two sampling methods used for collecting Ichneumonidae (Hymenoptera) in Cabañeros National Park (Spain). Eur. J. Entomol..

[39] Gibb H., Hochuli D.F. (2002). Habitat fragmentation in an urban environment: Large and small fragments support different arthropod assemblages. Biol. Conserv..

[40] Campbell J.W., Hanula J.L. (2007). Efficiency of Malaise traps and colored pan traps for collecting flower visiting insects from three forested ecosystems. J. Insect Conserv..

[41] Anderson M.J. (2001). A new method for non-parametric multivariate analysis of variance. Austral Ecol..

[42] Clarke K.R. (1993). Non-parametric multivariate analyses of changes in community structure. Austral J. Ecol..

[43] Hammer O., Harper D.A.T., Ryan P.D. (2001). Past: Paleontological Statistics software package for education and data analysis. Palaeontol. Electron..

[44] McCune B., Mefford M.J. (2016). PC-ORD. Multivariate Analysis of Ecological Data.

[45] Noyes J.S. (1989). A study of five methods of sampling Hymenoptera (Insecta) in a tropical rainforest, with special reference to the Parasitica. J. Nat. Hist..

[46] Wilson J.S., Griswold T., Messinger O.J. (2008). Sampling bee communities (Hymenoptera: Apiformes) in a desert landscape: Are pan traps sufficient?. J. Kansas Entomol. Soc..

[47] Duelli P., Obrist M.K., Schmatz D.R. (1999). Biodiversity evaluation in agricultural landscapes: Above-ground insects. Agric. Ecosyst. Environ..

[48] Pringle E.G., Dirzo R., Gordon D.M. (2012). Plant defense, herbivory, and the growth of *Cordia alliodora* trees and their symbiotic Azteca ant colonies. Oecologia.

[49] García J.L. (2003). Comparación de la captura de Hymenoptera (Insecta) mediante cuatro métodos de muestreo, en los cerros Yaví y Yutajé del Pantepui venezolano. Entomotropica.

[50] Machado A., Teixeira T., da Silva D., Fonseca M. (2012). Hymenoptera (Insecta: Hymenoptera) associated with silvopastoral systems. Agrofor. Syst..

[51] Barbosa M.C., Barbosa R., Henriques A.L. (2007). Perfil da fauna de vespas parasitóides (Insecta: Hymenoptera) em reserva florestal na Amazônia, Amazonas, Brasil. Entomotropica.

[52] Fernández F., Sharkey M.J. (2006). Introducción a Los Hymenoptera de la Región Neotropical.

[53] Mazón M. (2016). Taking shortcuts to measure species diversity: Parasitoid Hymenoptera subfamilies as surrogates of species richness. Biodivers. Conserv..

[54] García J.L., Montilla R. (2010). Hymenópteros parasitoides de insectos asociados a las plantaciones de cacao, en la región costera del estado Aragua, Venezuela. Agron. Trop..

[55] Anderson A., McCormack S., Helden A., Sheridan H., Kinsella A., Purvis G. (2011). The potential of parasitoid Hymenoptera as bioindicators of arthropod diversity in agricultural grasslands. J. Appl. Ecol..

[56] Narendran T.C., Upadhyay R.K., Mukerji K.G., Chamola B.P. (2001). Parasitic Hymenoptera and Biological Control. Biocontrol Potential and its Exploitation in Sustainable Agriculture.

[57] Kruess A., Tscharntke T. (1994). Habitat fragmentation, species loss and biological control. Science.

[58] Briceño A.J. (1997). Aproximación hacia un manejo integrado del barrenador de las meliaceas, *Hypsipyla grandella* (Zeller). Rev. For. Venez..

[59] Zaché B., Rodrigues da Costa R., Zanuncio J.C., Wilcken C.F. (2013). *Palmistichus elaeisis* (Hymenoptera, Eulophidae) parasitizing pupae of *Hypsipyla grandella* (Lepidoptera: Pyralidae). Fla. Entomol..

[60] Hernández F., Briceño A.J. (1999). Ciclo de vida del gusano esqueletizador *Eulepte gastralis* (Gn.) (Lepidoptera-Pyralidae), del apamate (*Tabebuia rosea* (Bertol.), DC.). Rev. For. Venez..

[61] Paul G.S., Montagnini F., Berlyn G.P., Craven D.J., van Breugel M., Hall J.S. (2012). Foliar herbivory and leaf traits of five native tree species in a young plantation of Central Panama. New For..

[62] Bos M.M., Steffan-Dewenter I., Tscharntke T. (2007). Shade tree management affects fruit abortion, insect pests and pathogens of cacao. Agric. Ecosyst. Environ..

[63] Ferronatto E.M.O. (1986). Preliminary observations on the host plants of the immature stages of the principal chrysomelids (Coleoptera: Chrysomelidae) occurring in cacao plantations. Rev. Theobroma.

[64] Konam J.K., Guest D.I. (2004). Role of beetles (Coleoptera: Scolytidae and Nitidulidae) in the spread of *Phytophthora palmivora* pod rot of cocoa in Papua New Guinea. Australas. Plant Pathol..

[65] Engelbrecht C.J., Harrington T.C., Alfenas A. (2007). Ceratocystis wilt of cacao—A disease of increasing importance. Phytopathology.

[66] Pérez-De la Cruz M., Equihua-Martínez A., Romero-Nápoles J., Sánchez-Soto S., García-López E. (2009). Diversidad, fluctuación poblacional y plantas huésped de escolitinos (Coleoptera: Curculionidae) asociados con el agroecosistema cacao en Tabasco, México. Rev. Mex. Biodivers..

[67] Novais S.M.A., Macedo-Reis L.E., Neves F.S. (2017). Predatory beetles in cacao agroforestry systems in Brazilian Atlantic forest: A test of the natural enemy hypothesis. Agrofor. Syst..

[68] Kreuger B., Potter D.A. (2001). Diet feeding activity and thermoregulation by Japanese beetles (Coleoptera: Scarabaeidae) within host plant canopies. Environ. Entomol..

[69] Plath M., Dorn S., Barrios H., Mody K. (2012). Diversity and composition of arboreal beetle assemblages in tropical pasture afforestations: Effects of planting schemes and tree species identity. Biodivers. Conserv..

[70] Bach C.E. (1981). Host plant growth form and diversity. Effects of abundance and feeding preference of a specialist herbivore, *Acalymma vittata* (Coleoptera: Chrysomelidae). Oecologia.

[71] Somarriba E. (1990). Sustainable timber production from uneven-aged shade stands of *Cordia alliodora* in small coffee farms. Agrofor. Syst..

[72] Navarro C., Montagnini F., Hernández G. (2004). Genetic variability of *Cedrela odorata* Linnaeus: Results of early performance of provenances and families from Mesoamerica grown in association with coffee. For. Ecol. Manag..

[73] Plath M., Mody K., Potvin C., Dorn S. (2011). Establishment of native tropical timber trees in monoculture and mixed-species plantations: Small-scale effects on tree performance and insect herbivory. For. Ecol. Manag..

[74] Araque O., Jaimez R.E., Azócar C., Espinoza W., Tezara W. (2009). Relaciones entre anatomía foliar, intercambio de gases y crecimiento en juveniles de cuatro especies forestales. Interciencia.

[75] Dauber J., Hirsch M., Simmering D., Waldhardt R., Otte A., Wolters V. (2003). Landscape structure as an indicator of biodiversity: Matrix effects on species richness. Agric. Ecosyst. Environ..

[76] Numa C., Verdú J.R., Sánchez A., Galante E. (2009). Effects of landscape structure on the spatial distribution of Mediterranean dung beetle diversity. Divers. Distrib..

[77] Inclán D.J., Cerretti P., Marini L. (2015). Landscape composition affects parasitoid spillover. Agric. Ecosyst. Environ..

[78] Plath M., Dorn S., Riedel J., Barrios H., Mody K. (2012). Associational resistance and associational susceptibility: Specialist herbivores show contrasting responses to tree stand diversification. Oecologia.

[79] Li Y., Weldegergis B.T., Chamontri S., Dicke M., Gols R. (2017). Does Aphid Infestation Interfere with Indirect Plant Defense against Lepidopteran Caterpillars in Wild Cabbage?. J. Chem. Ecol..

