# Supplementary material for: Entomofauna Associated with Agroforestry Systems of Timber Species and Cacao in the Southern Region of the Maracaibo Lake Basin (Mérida, Venezuela)
Source: Insects. 2018 Apr 20;9(2):46. doi: 10.3390/insects9020046 (PMC6023438; doi:10.3390/insects9020046)
Supplement: Supplementary file 1 [file insects-09-00046-s001.pdf]

Supplementary material

Table S1. Results obtained from SIMPER analysis when comparing those pairs of parasitoid Hymenoptera families assemblages with significant differences ($p < 0.05$). Av diss = Average of dissimilarity; Contrib % = % of contribution; Ced = *Cedrela odorata*; Cor = *Cordia thaisiana*; Swi = *Swietenia macrophylla*; Tab = *Tabebuia rosea*.

Ced-Swi			**Mean abundance**		*Ced-Tab*		**Mean abundance**	
**Family**	**Av diss**	**Contrib %**	**Ced**	**Swi**	**Av diss**	**Contrib %**	**Ced**	**Tab**
Diapriidae	4.859	12.09	2.05	1.62	5.03	11.92	2.05	2.14
Encyrtidae	4.819	11.98	1.78	2.48	5.168	12.24	1.78	2.78
Scelionidae	4.458	11.09	3.14	3.77	5.318	12.6	3.14	3.41
Braconidae	3.01	7.488	1.09	1.14	2.953	6.997	1.09	0.79
Ceraphronidae	2.887	7.182	0.861	0.788	2.704	6.405	0.861	0.559
Platygastridae	2.824	7.026	0.749	1.07	2.803	6.639	0.749	0.872
Mymaridae	2.723	6.773	2.32	2.42	3.483	8.252	2.32	2.41
Figitidae	2.705	6.73	0.777	1.03	2.764	6.548	0.777	1.28
Ichneumonidae	2.163	5.381	0.654	0.357	2.417	5.727	0.654	0.648
Pteromalidae	1.733	4.311	0.457	0.273	1.829	4.334	0.457	0.353
Chalcididae	1.633	4.062	0.101	0.489	1.124	2.662	0.101	0.267
Eucharitidae	1.509	3.754	0.339	0.326	1.645	3.897	0.339	0.381
Bethylidae	1.405	3.496	0.381	0.142	1.309	3.102	0.381	0.0833
Dryinidae	0.9521	2.369	0.243	0.0833	1.048	2.483	0.243	0.125
Eulophidae	0.9205	2.29	0.208	0.125	0.8012	1.898	0.208	0.0833
Eurytomidae	0.5402	1.344	0.0417	0.125	0.3559	0.8432	0.0417	0.0589
Trichogrammatidae	0.3775	0.9392	0	0.125	0.3707	0.8781	0	0.125
Evaniidae	0.2361	0.5873	0.0417	0.0417	0.1353	0.3206	0.0417	0
Eupelmidae	0.1937	0.482	0.0722	0	0.4263	1.01	0.0722	0.0833
Chrysididae	0.135	0.3359	0.0417	0	0.3348	0.7932	0.0417	0.0833
Signiphoridae	0.1168	0.2905	0	0.0417	0	0	0	0
Aphelinidae	0	0	0	0	0.1916	0.4539	0	0.0589

Cor-Swi			**Mean abundance**	
**Family**	**Av diss**	**Contrib %**	**Cor**	**Swi**
Diapriidae	4.869	12.92	2.57	1.62

Scelionidae	3.925	10.42	3.28	3.77
Encyrtidae	3.755	9.966	2.73	2.48
Mymaridae	2.772	7.358	2.49	2.42
Platygastridae	2.548	6.764	1.14	1.07
Ichneumonidae	2.537	6.733	0.932	0.357
Figitidae	2.517	6.681	0.928	1.03
Ceraphronidae	2.511	6.665	0.901	0.788
Braconidae	2.236	5.935	1.03	1.14
Pteromalidae	2.01	5.336	0.672	0.273
Eucharitidae	1.657	4.398	0.475	0.326
Chalcididae	1.586	4.21	0.256	0.489
Eulophidae	1.329	3.526	0.451	0.125
Bethylidae	1.101	2.923	0.298	0.142
Trichogrammatidae	0.4825	1.281	0.0722	0.125
Dryinidae	0.4532	1.203	0.0833	0.0833
Eurytomidae	0.3989	1.059	0	0.125
Aphelinidae	0.2395	0.6358	0.101	0
Evaniidae	0.2279	0.6051	0.0417	0.0417
Eupelmidae	0.2058	0.5462	0.0833	0
Signiphoridae	0.1926	0.5111	0.0417	0.0417
Chrysididae	0.1214	0.3223	0.0417	0

Table S2. Results obtained from SIMPER analysis when comparing those pairs of Coleoptera families assemblages with significant differences ($p < 0.05$) amongst timber trees. Av diss = Average of dissimilarity; Contrib % = % of contribution; Cor = *Cordia thaisiana*; Tab = *Tabebuia rosea*.

Cor-Tab			Mean abundance	
**Family**	**Av diss**	**Contrib %**	**Cor**	**Tab**
Chrysomelidae	11.21	20.44	3.28	2.30
Scarabaeidae	7.26	13.24	0.954	1.24
Coccinellidae	6.338	11.56	0.9	0.623
Elateridae	5.693	10.38	0.719	0.483
Staphylinidae	2.363	4.309	0.0417	0.309
Curculionidae	3.89	7.095	0.302	0.485
Aphodiidae	2.916	5.318	0.156	0.397
Scydmaenidae	2.765	5.043	0.267	0.25
Biphyllidae	1.242	2.264	0.0589	0.167
Hybosoridae	0.7051	1.286	0.0417	0.0833
Histeridae	0.7296	1.331	0	0.101
Mordellidae	1.904	3.472	0.235	0.0833
Pselaphidae	0.3966	0.7233	0	0.0417
Carabidae	0.9643	1.759	0.0417	0.0833
Dytiscidae	0.6417	1.171	0	0.101
Erotylidae	0.6396	1.166	0.0833	0
Cicindelidae	0.5749	1.048	0	0.0833
Anthicidae	0.4884	0.8906	0.0833	0
Rutelidae	0.2095	0.382	0.0417	0
Lycidae	1.471	2.683	0.214	0
Lampyridae	0	0	0	0
Colydiidae	0.2963	0.5403	0.0589	0
Rhizophagidae	0	0	0	0
Mycetophagidae	0	0	0	0
Meloidae	0	0	0	0

Table S3. Results obtained from SIMPER analysis when comparing those pairs of Coleoptera families assemblages with significant differences ($p < 0.05$) amongst cacao cultivars. Av diss = Average of dissimilarity; Contrib % = % of contribution; Guas = Guasare; Lob = Lobatera; Mer = Merideño.

Guas-Lob			**Mean abundance**		*Guas-Mer*		**Mean abundance**	
**Family**	**Av diss**	**Contrib %**	**Guas**	**Lob**	**Av diss**	**Contrib %**	**Guas**	**Mer**
Chrysomelidae	10.2	18.64	2.39	2.75	8.57	16.57	2.39	2.76
Scarabaeidae	8.777	16.05	1.08	1.52	9.877	19.09	1.08	2
Coccinellidae	6.447	11.79	0.648	0.847	6.287	12.15	0.648	0.969
Elateridae	6.163	11.27	0.454	0.79	5.382	10.4	0.454	0.755
Staphylinidae	2.777	5.077	0.0833	0.359	1.187	2.296	0.0833	0.125
Curculionidae	3.869	7.073	0.243	0.461	3.678	7.111	0.243	0.476
Aphodiidae	1.604	2.933	0.203	0.0589	2.2	4.254	0.203	0.156
Scydmaenidae	4.031	7.37	0.167	0.51	2.763	5.341	0.167	0.292
Biphyllidae	1.599	2.923	0	0.25	1.109	2.144	0	0.201
Hybosoridae	0.2362	0.4319	0	0.0417	0.7628	1.475	0	0.114
Histeridae	0.5045	0.9224	0.0417	0.0417	0.7677	1.484	0.0417	0.0589
Mordellidae	1.304	2.384	0.0417	0.177	1.485	2.871	0.0417	0.226
Pselaphidae	0.7035	1.286	0.101	0	0.9304	1.799	0.101	0.0417
Carabidae	0.6932	1.267	0.0417	0.0417	0.2674	0.517	0.0417	0
Dytiscidae	0	0	0	0	0.725	1.402	0	0.101
Erotylidae	1.031	1.884	0.0833	0.0417	0.6998	1.353	0.0833	0
Cicindelidae	0.2362	0.4319	0	0.0417	0	0	0	0
Anthicidae	0.5252	0.9603	0.0417	0.0589	0.9476	1.832	0.0417	0.125
Rutelidae	0.2466	0.4508	0	0.0417	0.2206	0.4265	0	0.0417
Lycidae	0.8874	1.622	0.0417	0.101	0.5034	0.9731	0.0417	0.0417
Lampyridae	0.2236	0.4088	0	0.0417	0	0	0	0
Colydiidae	0.2772	0.5068	0	0.0417	0.504	0.9744	0	0.101
Rhizophagidae	0	0	0	0	0.192	0.3712	0	0.0417
Mycetophagidae	0	0	0	0	0.222	0.4293	0	0.0417
